# Numerical study of nano-biofilm stagnation flow from a nonlinear stretching/shrinking surface with variable nanofluid and bioconvection transport properties

**DOI:** 10.1038/s41598-021-88935-9

**Published:** 2021-05-10

**Authors:** Abdulaziz Alsenafi, O. Anwar Bég, M. Ferdows, Tasveer A. Bég, A. Kadir

**Affiliations:** 1 Department of Mathematics, Kuwait University, Kuwait City, Kuwait; 2 Department of Mechanical/Aeronautical Engineering, Salford University, Manchester, M54WT UK; 3 Research Group of Fluid Flow Modeling and Simulation, Department of Applied Mathematics, University of Dhaka, Dhaka, Bangladesh; 4 Renewable Energy and Computational Multi-Physics, Israfil House, Dickenson Rd., Manchester, M13 UK

**Keywords:** Engineering, Mathematics and computing, Nanoscience and technology, Physics

## Abstract

A mathematical model is developed for stagnation point flow toward a stretching or shrinking sheet of liquid nano-biofilm containing spherical nano-particles and bioconvecting gyrotactic micro-organisms. Variable transport properties of the liquid (viscosity, thermal conductivity, nano-particle species diffusivity) and micro-organisms (species diffusivity) are considered. Buongiorno’s two-component nanoscale model is deployed and spherical nanoparticles in a dilute nanofluid considered. Using a similarity transformation, the nonlinear systems of partial differential equations is converted into nonlinear ordinary differential equations. These resulting equations are solved numerically using a central space finite difference method in the CodeBlocks Fortran platform. Graphical plots for the distribution of reduced skin friction coefficient, reduced Nusselt number, reduced Sherwood number and the reduced local density of the motile microorganisms as well as the velocity, temperature, nanoparticle volume fraction and the density of motile microorganisms are presented for the influence of wall velocity power-law index (*m*), viscosity parameter $$({c}_{2})$$, thermal conductivity parameter (*c*_*4*_), nano-particle mass diffusivity (*c*_*6*_), micro-organism species diffusivity (*c*_*8*_), thermophoresis parameter $$(Nt)$$, Brownian motion parameter $$(Nb)$$, Lewis number $$(Le)$$, bioconvection Schmidt number $$(Sc)$$, bioconvection constant (*σ*) and bioconvection Péclet number $$(Pe)$$. Validation of the solutions via comparison related to previous simpler models is included. Further verification of the general model is conducted with the Adomian decomposition method (ADM). Extensive interpretation of the physics is included. Skin friction is elevated with viscosity parameter ($${\mathrm{c}}_{2})$$ whereas it is suppressed with greater Lewis number and thermophoresis parameter. Temperatures are elevated with increasing thermal conductivity parameter ($${\mathrm{c}}_{4})$$ whereas Nusselt numbers are reduced. Nano-particle volume fraction (concentration) is enhanced with increasing nano-particle mass diffusivity parameter ($${c}_{6}$$) whereas it is markedly reduced with greater Lewis number (*Le*) and Brownian motion parameter (*Nb*). With increasing stretching/shrinking velocity power-law exponent ($$m),$$ skin friction is decreased whereas Nusselt number and Sherwood number are both elevated. Motile microorganism density is boosted strongly with increasing micro-organism diffusivity parameter ($${\mathrm{c}}_{8}$$) and Brownian motion parameter (*Nb*) but reduced considerably with greater bioconvection Schmidt number (*Sc*) and bioconvection Péclet number (*Pe*). The simulations find applications in deposition processes in nano-bio-coating manufacturing processes.

## Introduction

Stagnation point flows constitute an important category of problems in modern fluid mechanics. They are characterized by flows in the immediate vicinity of a solid surface at which the impinging fluid bifurcates into different streams. At the impingement point the flow velocity vanishes i.e. the fluid stream stagnates due to the no-slip condition. Many types of stagnation flow have been studied since they are a special case of the Navier–Stokes equations and therefore provide a convenient framework for boundary-layer solutions to be derived^1^. These include plane (orthogonal) stagnation point flow, axisymmetric stagnation flow (impinging jet or symmetrical radial flow) and non-orthogonal (oblique) stagnation flow. Stagnation flows are also often termed Hiemenz flows. In recent years numerous studies of such flows have been presented in many intriguing applications spanning geophysics, manufacturing and transport systems. These include aerosol deposition of thin films^2^, meteorological aerodynamics^3^, flame extinction scenarios in near-limit premixed combustion for rocket propulsion^4^, laser drilling transport phenomena^5^, smart solar coating synthesis^6^ and impinging jet chemical vapor deposition (CVD) reactors^7^. In process mechanical/chemical engineering operations, frequently the surface may also be *expanding* or *contracting* in order to modify coating properties. Mahapatra and Gupta^8^ studied axisymmetric stagnation‐point Newtonian flow and convection towards a stretching surface, observing that a boundary‐layer structure is generated if the free stream velocity exceeds the stretching velocity of the surface whereas an inverted boundary layer structure is created for the reverse case. They also showed that heat is transferred significantly from the wall to the zone surrounding the stagnation point when wall temperature is greater than ambient temperature.

Increasingly in recent years engineers have adopted bio-inspired mechanisms in technological designs. These developments have been driven by the desire to achieve more adaptive, intelligent systems which require less maintenance and attain significant improvements in durability and also ecological compatibility (“green” systems). Many different aspects have been studied including surface tension, compliance (flexibility which affects boundary layer drag), natural adhesion, hydrophobic, self-healing, anti-fouling and internal architecture modification of materials via doping with complex biological species^9^. Although biofilms (e.g. denitrifying and oxic) are generally undesirable in engineering systems (since they involve deposition of surface-attacking bacteria)^10^, new technologies are being developed which can mitigate this phenomenon via the incorporation of specific bio-species, enzymes, cohesive agents etc.^11,12^. A new generation of biohybrid thin film coatings has emerged which feature stretchable and thermally conductive bio-interfaces^13^ which exploit unique micro-organisms. Many intricate mechanisms of motion have been identified in such biological species. A key pattern of behavior which has been studied in recent years is *biconvection*^14^. This refers to a random pattern formation in suspensions of micro-organisms due to swimming of the microorganisms in aqueous media. Generally, the microorganisms possess marginally greater densities than water and invariably they propel in an upward direction against gravity. on average they swim upwards although the reasons for *up-swimming* may be different for different species. Different families of micro-organisms respond to different external stimuli and this controls the propulsion direction. These stimuli produce taxes i.e. reactive behavior to the stimulus. Many different taxes have been observed including *photo-tactic* *bioconvection* (response to light)^15^ (the associated single-celled algae show some potential in photo-voltaic coatings), *chemo-tactic* *bioconvection* (where chemical concentration, acidity, alkalinity, etc. are the controlling stimuli)^16^, *oxy-tactic* *bioconvection* (oxygen is the stimulus), *electro-tactic* *bioconvection* (electrical fields manipulate microorganisms such as zoospores in phytopathogenic fungi)^17^, *geo-tactic* *bioconvection* (vibration is the stimulus)^18^, *gravi-tactic* *bioconvection* (micro-organisms swim away from gravity effects) and *gyro-tactic* *bioconvection*^19^. In the last of these types (gyrotaxis), swimming of microorganisms is controlled by the balance between the torque due to gravity acting on a bottom-heavy cell and the torque due to viscous forces arising from local shear flows. The continuum model developed in Ref.^19^ has allowed engineers to further explore the dynamics of *gyro-tactic* *bioconvection* in which the species are non-spherical, and their orientation is influenced by the strain rate in the ambient flow as well as the vorticity. Important recent studies of mathematical modelling of gyrotactic bioconvection (of relevance to biofuel systems, drug delivery and bio-coatings etc.) include Kuznetsov and Avramenko^20^ who conducted a linear stability analysis of a shallow layer of suspended micro-organisms and determined that critical Rayleigh number is elevated with increasing density of particles and that this aids in stabilizing the suspensions. Gyrotactic axisymmetric bioconvection plumes were examined by Geng and Kuznetsov^21^ who considered in detail the entrainment of gyrotactic micro-organisms during the sedimentation process. They also established that bioconvection may be simulated as a two-phase flow process in which the micro-organisms represent the “solid” phase, but distinct from conventional multi-phase flows, the micro-organisms are self-propelling. Geng and Kuznetsov^22^ further investigated the biconvection in a bi-dispersed suspension of small solid particles that have different densities and settling velocities in a fluid that contains motile gyrotactic micro-organisms, also considering Brownian diffusion. These studies also revealed that when micro-organisms are heavier towards the rear, gyrotaxis re-directs them such that they swim towards regions of most rapid downflow and this may lead to bioconvective instability can develop from an initially uniform suspension, without an unstable density stratification. An important class of gyrotactic micro-organisms is algal species (*Chlamydomonas* *nivalis*) which has been explored in bio-coatings^11,12^.

- set of rules, $R'_i:$
  i. for each rule of type (1), $E/a^c \rightarrow a^p \in R_i,$ if $(i,j_1),\ldots (i,j_h)\in syn,$ $h\ge 1,$ a rule $a^c\rightarrow (a^p,C_{j_1},) \ldots , (a^p,C_{j_h})\ \{g_E\}$ is added to $R'_i$, where $g_E$ is the guard obtained from the regular expression *E*. Please note that as $(i,j_1),\ldots (i,j_h)\in syn,$ then one must have $\{i,j_1\},\ldots \{i,j_h\}\in L.$ When $h=0,$ i.e., no synapse going out of $\sigma _i$, then the corresponding rule in $R'_i$ is $a^c\rightarrow (a^p,Env)\ \{g_E\}.$ $R'_{Env},$ the set of rules associated with type *Env*,  is $\emptyset .$
  ii. for each rule of type (2), $a^c \rightarrow \lambda \in R_i,$ a rule $a^c\rightarrow \lambda \ \{=a^c\}$ is added to $R'_i.$
- execution strategy, $\delta _i:$ is always choice, as one single rule from those applicable must be selected; $\delta _{{\text {Env}}}$ is also choice.

A significant development in twenty-first century engineering has been the emergence of nanomaterials. Engineers are increasingly designing systems at the nanoscale and important progress has been made in nanotube-embedded gels, nano-lubricants, electro-conductive nano-polymers etc. An important sub-group of liquid nanomaterials is *nanofluids*. Introduced in the 1990s by Choi et al*.*^23^, these complex fluids were developed primarily to achieve substantial improvements in thermal enhancement. They are synthesized by doping conventional working fluids e.g. water, polymers, ethylene glycol etc., with either metallic or carbon-based nanoparticles with average particle sizes below 100 nm. The resulting colloidal suspension achieves superior thermal conductivity, heat capacity and viscosity properties compared with macroscopic fluids. An impressive range of industrial sectors have embraced nanofluid technology including nuclear reactor cooling^24^, aerospace and naval lubricants^25^, polymer coating processes^26^, pharmacodynamics^27^ (where targeted drug delivery can be achieved via precision engineered nano-particles introduced into the blood stream), direct absorber solar collectors^28^, spin coating of rocket structures for enhanced thermal protection^29^, medical lubrication^30^ and petroleum extraction processes^31^. Computational nanofluid dynamics has also received significant attention in the past decade. Many different models have been employed with a variety of numerical schemes to solve the complex differential equation systems required to simulate nanoscale transport phenomena. For example, Kumar et al.^32^ used the Tiwari-Das volume fraction nanofluid model and MATLAB quadrature to compute heat and mass transfer rates in time-dependent magnetite nanofluid flow from a stretched nano-coating. They considered rheological effects using the Stokes’ couple stress microstructural model and also considered Joule heating effects for copper and aluminium oxide metallic nano-particles. Engineers have also explored with some vigour the efficiency of nano-doped polymeric coatings as multi-functional smart materials in environments featuring significant corrosion, thermal loading and abrasion. Interesting studies in this regard include Aliofkhazraei^33^. Stretchable nanofluid coatings have been investigated by Yao and Zhu^34^. Zirconium oxide doped nanofluid coatings have been explored as sensors for various stimuli including strain, heat and ultraviolet radiation^35^. Quite recently engineers have begun to combine biological phenomena and nanofluid physics since they provide a dual benefit and produce yet more intelligent materials for ever-increasing applications. Javid et al*.*^36^ considered the used o nanoparticle doped titanium oxide film coatings for anti-bacterial protection. Kuznetsov^37^ first investigated bioconvection in nanofluid transport where both gyrotactic and oxytactic micro-organisms were studied and significant improvements in heat and mass transfer were achieved by simultaneous use of nano-particles and non-interacting swimming bio-species. Balla et al*.*^38^ employed a finite volume computational method to simulate oxytactic bioconvection in an enclosure containing a nanofluid-saturated porous medium. They observed that increasing bioconvection Péclet number and bioconvection Rayleigh number in addition to thermophoresis assist the flow whereas the opposite influence is computed with increasing Brownian motion and bioconvection Lewis numbers. Vasu et al*.*^39^ used homotopy and generalized differential quadrature to study transient hydromagnetic viscoplastic bio-nanocoating stretching flow doped with metallic magnetic nano-particles and gyrotactic micro-organisms. As noted earlier, *stagnation-point* *nanofluid* *flows* are of considerable relevance to nano-materials processing technologies. These have also been investigated in detail in recent years both with and without bioconvection. Uddin et al*.*^40^ used MAPLE symbolic software to simulate the stagnation point nanofluid gyrotactic bioconvection coating flow from a translating sheet with multiple slip (hydrodynamic, thermal and species) effects. Shukla et al*.*^41^ applied homotopy and finite element method to compute the entropy generation in transient nanofluid stagnation-point flow from an extending sheet with homogenous chemical reaction, radiative heat flux, magnetic field, electrical field and wall transpiration effects. Kumar and Sood^42^ employed Keller’s finite difference box method to simulate the bioconvection induced by unsteady stagnation-point flow of a magnetized nano-liquid stretching sheet containing gyrotactic suspension of microorganisms and computed extensive results for local skin friction, Nusselt number, Sherwood number and wall gradient density number of microorganisms. These nanofluid bioconvection modelling studies however did not consider *variable* *transport* properties which are known to arise in real applications where agglomeration of nano-particles and clustering of micro-organisms may arise^11,12^. Kang et al*.*^43^ used a molecular dynamics method to analyze the influence of nanoparticle aggregation on thermal conductivity and viscosity of nanofluids with a Green–Kubo formulation. They identified that clustering of nanoparticles produces strong elevation in thermal conductivity in nanofluid whereas it induces a weaker enhancement in viscosity and that the nature of clustering also exerts an influence on these properties. Sahoo et al*.*^44^ presented extensive laboratory results for thermal conductivity variation in silicon dioxide (SiO_2_) nanoparticles dispersed in 60% ethylene glycol and 40% water base fluids. They observed that over a temperature range of 20 °C to 90 °C and for several particle volumetric concentrations of up to 10%, there is an elevation in ratio of thermal conductivity of nanofluid to that of the base fluid with greater temperature and volumetric concentration. Begum et al.^45^ studied numerically the impact of variable thermophysical properties on gyrotactic bioconvection nanofluid boundary layer flow along a uniformly heated vertical cone with Buongiorno’s nanoscale model, noting that variable thermophysical properties elevate wall heat transfer rates compared with constant properties.

In the present article, a mathematical model is developed for stagnation point flow towards a stretching or shrinking sheet of liquid nano-biofilm coating containing spherical nano-particles and bioconvecting gyrotactic micro-organisms. Buongiorno’s two-component nanoscale model is deployed and spherical nanoparticles in a dilute nanofluid considered Variable transport properties of the liquid (viscosity, thermal conductivity, nano-particle species diffusivity) and micro-organisms (species diffusivity) are examined. Explicit formulations for the variable properties are described. The transformed dimensionless steady-state boundary layer conservation equations for mass, momentum, heat, nano-particle concentration and motile microorganism density number, which amount to a coupled system of nonlinear ordinary differential equations with associated boundary conditions, are solved computationally with a central space finite difference method in the Code Blocks Fortran platform^46,47^. Graphical plots for the distribution of reduced skin friction coefficient, reduced Nusselt number, reduced Sherwood number and the reduced local density of the motile microorganisms as well as the velocity, temperature, nanoparticle volume fraction and the density of motile microorganisms are presented for the influence of wall velocity power-law index (*m*), viscosity parameter $({c}_{2})$, thermal conductivity parameter (*c*_*4*_), nano-particle mass diffusivity (*c*_*6*_), micro-organism species diffusivity (*c*_*8*_), thermophoresis parameter $(Nt)$, Brownian motion parameter $(Nb)$, Lewis number $(Le)$, bioconvection Schmidt number $(Sc)$, bioconvection constant (*σ*) and bioconvection Péclet number $(Pe)$. Validation of the solutions via comparison related to earlier published results in the literature is included. Further verification of the general model is conducted with the Adomian decomposition method (ADM)^48^. Detailed elaboration of the physics is provided. The present study constitutes a novel contribution to bioconvection nanoliquid coating analysis and simulation.

## Mathematical model for bioconvection nanofluid coating flow

**Figure 1:**
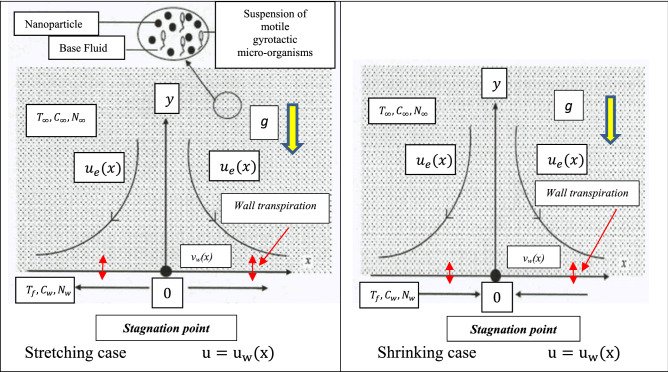
Physical model and coordinate system for stagnation stretching/shrinking bio-nanocoating flow.

The coating model under investigation comprises steady, two-dimensional flow of an incompressible nanofluid containing gyrotactic bioconvecting micro-organisms and spherical nanoparticles (which do not interact), in the region $y > 0$ driven by a permeable stretching/shrinking surface located at $y = 0$ with a fixed stagnation point at $x = 0$ as shown in Fig. 1. Buongiorno’s two-component nanoscale model is deployed and spherical nanoparticles in a dilute nanofluid considered. The stretching/shrinking velocity $u_{w} \left( x \right)$ and the ambient fluid velocity $u_{e} \left( x \right)$ are assumed to vary as $u_{w} \left( x \right) = cx^{m}$ and $u = u_{e} \left( x \right) = ax^{m} { }$, where $a, c$ and $m$ are constants with $a > 0$ and $m > 0$. Wall transpiration (suction/blowing) is also incorporated via the vertical velocity component,${ }v_{w} \left( x \right)$*.* The parameter *m* denotes the *power-law* *velocity* *exponent*; $m = 1$ corresponds to the *linear* case and $m > 1$ is associated with the *non-linear* case. The case *m* = 0 implies *no* *stretching* *or* *shrinking* *of* *the* *sheet* i.e. a fixed sheet. It is also noteworthy that ${\text{c}} > 0$ and ${\text{c}} < 0$ correspond to *stretching* and *shrinking* sheets, respectively. O*rthogonal* (*90* *degrees* *impingement*) *coating* *stagnation* *point* *flow* of a substrate is considered but the sheet coating may be stretched or contracted depending on the process being deployed in industry.

Following Kuznetsov and Nield^49^ and Zaimi et al*.*^50^, uniform temperature $\left( {T_{w} } \right)$, uniform nanofluid volume fraction $(C_{w} )$ and uniform concentration (number density) of motile microorganisms ($N_{w}$), are considered at the surface of the sheet are. Also, uniform temperature ($T_{\infty }$), uniform nanofluid volume fraction $(C_{\infty }$) and uniform concentration (number density) of motile micro-organisms $\left( {N_{\infty } } \right)$ are prescribed far from the surface of the sheet i.e. in the free stream. A dilute nanofluid is considered and agglomeration effects neglected. Under the above assumptions, the governing equations for conservation of mass, momentum, thermal energy, nanoparticle volume fraction and motile microorganism density, can be written, by extending the model of Ref.^50^ to incorporate variable properties (see Amirsom et al*.*^51^), as:

1 $$\frac{{\partial {\text{u}}}}{{\partial {\text{x}}}} + \frac{{\partial {\text{v}}}}{{\partial {\text{y}}}} = 0,$$

2 $${\text{u}}\frac{{\partial {\text{u}}}}{{\partial {\text{x}}}} + {\text{ v}}\frac{{\partial {\text{u}}}}{{\partial {\text{y}}}} = {\text{u}}_{{\text{e}}} \frac{{{\text{du}}_{{\text{e}}} }}{{{\text{dx}}}} + \left( {\frac{1}{{{\uprho }_{\infty } }}} \right)\frac{\partial }{{\partial {\text{y}}}}\left[ {{\upmu }\left( {\text{C}} \right)\frac{{\partial {\text{u}}}}{{\partial {\text{y}}}}} \right],$$

3 $${\text{u}}\frac{{\partial {\text{T}}}}{{\partial {\text{x}}}} + {\text{v}}\frac{{\partial {\text{T}}}}{{\partial {\text{y}}}} = \frac{1}{{{\uprho }_{\infty } {\text{c}}_{{\text{p}}} }}{ }\frac{\partial }{{\partial {\text{y}}}}\left[ {{\text{k}}\left( {\text{C}} \right)\frac{{\partial {\text{T}}}}{{\partial {\text{y}}}}} \right] + {\uptau }\frac{\partial }{{\partial {\text{y}}}}\left[ {{\text{D}}_{{\text{B}}} \left( {\text{C}} \right)\left( {{\text{C}} - {\text{C}}_{\infty } } \right)} \right]\frac{{\partial {\text{T}}}}{{\partial {\text{y}}}} + {\uptau }\frac{{{\text{D}}_{{\text{T}}} }}{{{\text{T}}_{{{ }\infty }} }}\left( {\frac{{\partial {\text{T}}}}{{\partial {\text{y}}}}} \right)^{2} ,$$

4 $${\text{u}}\frac{{\partial {\text{C}}}}{{\partial {\text{x}}}} + {\text{v}}\frac{{\partial {\text{C}}}}{{\partial {\text{y}}}} = \frac{\partial }{{\partial {\text{y}}}}\left[ {{\text{D}}_{{\text{B}}} \left( {\text{C}} \right)\frac{{\partial {\text{C}}}}{{\partial {\text{y}}}}} \right]{ } + \frac{{{\text{D}}_{{\text{T}}} }}{{{\text{T}}_{\infty } }}\frac{{\partial^{2} {\text{T}}}}{{\partial {\text{y}}^{2} }},$$

5 $${\text{u}}\frac{{\partial {\text{n}}}}{{\partial {\text{x}}}} + {\text{v}}\frac{{\partial {\text{n}}}}{{\partial {\text{y}}}} + \frac{{{\text{bW}}_{{\text{c}}} }}{{\Delta {\text{C}}_{{\text{w}}} }}\left[ {\frac{\partial }{{\partial {\text{y}}}}\left( {{\text{n}}\frac{{\partial {\text{C}}}}{{\partial {\text{y}}}}} \right)} \right] = \frac{\partial }{{\partial {\text{y}}}}\left[ {{\text{D}}_{{\text{n}}} \left( {\text{C}} \right)\frac{{\partial {\text{n}}}}{{\partial {\text{y}}}}} \right].$$

The prescribed boundary conditions at the wall (sheet) and free stream^50^ are:

$$\left\{ {\begin{array}{*{20}c} {u = {\text{u}}_{{\text{w}}} \left( {\text{x}} \right) = c{\text{x}}^{{\text{m}}} , \,v = {\text{v}}_{{\text{w}}} \left( {\text{x}} \right) = - \frac{{{\text{m}} + 1}}{2}\sqrt {\frac{{{\text{u}}_{{\text{e}}} \left( {\text{x}} \right){\upnu }_{\infty } }}{{\text{x}}}} S, } \\ {T = {\text{T}}_{{\text{f}}} ,\,C = {\text{C}}_{{\text{w}}} ,\,{\text{n}} = {\text{n}}_{{\text{w}}} } \\ \end{array} } \right.;\,\,\,\,{\text{at}}\,\,{\text{y}} = 0{ ,}$$

6 $${\text{u}} \to {\text{u}}_{{\text{e}}} \left( {\text{x}} \right) = {\text{ax}}^{{\text{m}}} { },\,\,{\text{T}} \to {\text{T}}_{\infty } { },\,\,{\text{C}} \to {\text{C}}_{\infty } { },\,\,{\text{n}} \to {\text{n}}_{{\infty { }}} ;\quad {\text{as}}\,\,{\text{y}} \to \infty .$$

In the above equations, the following notation applies: $\left( {u, v} \right)$ are the nanofluid velocity components, $T$ is the nanofluid temperature, $C$ is the nano-particle concentration (volume fraction), $n$ is the density of motile gyrotactic micro-organisms, $u_{e} \left( x \right)$ is the dimensional external fluid velocity, $\rho_{\infty }$ is the constant fluid density, $c_{p}$ is the specific heat at constant pressure, $b$ is the chemotaxis constant, $W_{c}$ is the maximum cell swimming speed, $\mu \left( C \right)$ is the variable dynamic viscosity, $k\left( C \right)$ is the variable thermal conductivity, $D_{B} \left( C \right)$ is the variable mass diffusivity of nano-particles (variable Brownian diffusion coefficient), $D_{n} \left( C \right)$ is the variable diffusivity of gyrotactic micro-organisms, $D_{T}$ is the thermophoretic diffusion coefficient, $\tau = \left( {\rho c} \right)_{p} /\left( {\rho c} \right)_{f}$ is the ratio of effective heat capacity of the nanoparticle material to the heat capacity of the base fluid (water), $\mu_{\infty }$ is the constant dynamic viscosity, $k_{\infty }$ is the constant thermal conductivity, $D_{B,\infty }$ is the constant nano-particle mass diffusivity, $D_{n,\infty }$ is the constant micro-organism diffusivity, $c_{2}$ is the dimensionless viscosity parameter, $c_{4}$ is the thermal conductivity parameter, ${\text{c}}_{6}$ is the mass diffusivity parameter, $c_{8}$ is the micro-organism diffusivity parameter (Amirsom et al*.*^51^). Furthermore, *m* is the stretching/shrinking velocity power-law exponent. Proceeding with the analysis, it is advantageous to introduce the dimensionless functions $f, \theta \phi \,{\text{ and}}\, \chi$ (for dimensionless stream function, temperature, nano-particle volume fraction and motile micro-organism density number) in order to convert the governing partial differential equations into ordinary differential equations with regard to a similarity variable, $\eta$ (dimensionless transverse coordinate) as follows^50,51^:

7 $${\upeta } = {\text{y}}\sqrt {\frac{{{\text{u}}_{{\text{e}}} \left( {\text{x}} \right)}}{{{\upnu }_{\infty } {\text{x}}}}{ }} ,$$

8 $${\uppsi } = \sqrt {{\text{u}}_{{\text{e}}} \left( {\text{x}} \right){\upnu }_{\infty } {\text{x}}} {\text{ f}}\left( {\upeta } \right){ ,}$$

9 $${\uptheta }\left( {\upeta } \right) = \frac{{{\text{T}} - {\text{T}}_{\infty } }}{{{\text{T}}_{{\text{f}}} - {\text{T}}_{\infty } }}{ ,}$$

10 $$\phi \left( {\upeta } \right) = \frac{{{\text{C}} - {\text{C}}_{\infty } }}{{{\text{C}}_{{\text{w}}} - {\text{C}}_{\infty } }},$$

11 $${\upchi }\left( {\upeta } \right) = \frac{{{\text{n}} - {\text{n}}_{\infty } }}{{{\text{n}}_{{\text{w}}} - {\text{n}}_{\infty } }},$$

12 $${\upmu }\left( {\text{C}} \right) = {\upmu }_{\infty } \left[ {1 + {\text{c}}_{1} \left( {{\text{C}} - {\text{C}}_{\infty } } \right)} \right] = {\upmu }_{\infty } \left[ {1 + {\text{c}}_{2} \phi \left( {\upeta } \right)} \right]{,}$$

13 $${\text{k}}\left( {\text{C}} \right) = {\text{k}}_{\infty } \left[ {1 + {\text{c}}_{3} \left( {{\text{C}} - {\text{C}}_{\infty } } \right)} \right] = {\text{k}}_{\infty } \left[ {1 + {\text{c}}_{4} \phi \left( {\upeta } \right)} \right],$$

14 $${\text{D}}_{{\text{B}}} \left( {\text{C}} \right) = {\text{D}}_{{{\text{B}},\infty }} \left[ {1 + {\text{c}}_{5} \left( {{\text{C}} - {\text{C}}_{\infty } } \right)} \right] = {\text{D}}_{{{\text{B}},\infty }} \left[ {1 + {\text{c}}_{6} \phi \left( {\upeta } \right)} \right],$$

15 $${\text{D}}_{{\text{n}}} \left( {\text{C}} \right) = {\text{D}}_{{{\text{n}},\infty }} \left[ {1 + {\text{c}}_{7} \left( {{\text{C}} - {\text{C}}_{\infty } } \right)} \right] = {\text{D}}_{{{\text{n}},\infty }} \left[ {1 + {\text{c}}_{8} \phi \left( {\upeta } \right)} \right].$$

Here $\psi$ is the dimensional stream function. Thus Eqs. (2)–(5) reduce to the following non-dimensional similarity differential equations:

16 $$\left( {1 + {\text{c}}_{2} \phi } \right){\text{f}}^{\prime\prime\prime} + {\text{c}}_{2} {\text{f}}^{\prime\prime}\phi^{\prime} + \frac{{{\text{m}} + 1}}{2}{\text{ff}}^{\prime\prime} - {\text{mf}}^{\prime 2} + {\text{m}} = 0,$$

17 $$(1 + {\text{c}}_{4} \phi ){\uptheta }^{{^{\prime\prime}}} + {\text{Pr}}\frac{{{\text{m}} + 1}}{2}{\text{f}}\theta^{\prime} + {\text{c}}_{4} {{\theta^{\prime}}}\phi^{\prime} + {\text{Nb}}\left[ {1 + 2{\text{c}}_{6} \phi } \right]{{\theta^{\prime}}}\phi^{\prime} + {\text{Nt}}\theta^{\prime{2}} = 0,$$

18 $$\left( {1 + {\text{c}}_{6} \phi } \right)\phi^{\prime\prime} + {\text{Le}}\frac{{{\text{m}} + 1}}{2}{\text{f}}\phi^{\prime} + {\text{c}}_{6} \phi^{{{^{\prime}}2}} + \frac{{{\text{Nt}}}}{{{\text{Nb}}}}{{\theta^{\prime\prime}}} = 0,$$

19 $$\left( {1 + {\text{c}}_{8} \phi } \right){{\chi^{\prime\prime}}} + {\text{Sc}}\frac{{{\text{m}} + 1}}{2}{\text{f}{\chi^{\prime}}} + {\text{c}}_{8} \phi^{\prime}{{\chi^{\prime}}} - {\text{Pe}}\left[ {\phi^{\prime}{{\chi^{\prime}}} + \phi^{\prime\prime}\left( {{\upsigma } + {\upchi }} \right)} \right] = 0{ }{\text{.}}$$

The emerging dimensionless boundary conditions become:

$${\text{f}}\left( 0 \right) = {\text{S}},{\text{ f}^{{\prime}}}\left( 0 \right) = {\uplambda },\,{{ \theta }}\left( 0 \right) = 1,\,{ }\phi \left( 0 \right) = 1,\,{{ \chi }}\left( 0 \right) = 1,$$

20 $${\text{f}^{{\prime}}}\left( \infty \right) \to 1,\,{{ \theta }}\left( \infty \right) \to 0,\,{ }\phi \left( \infty \right) \to 0,\,{{ \chi }}\left( \infty \right) \to 0.$$

Here, $\lambda \left( { = \frac{{\text{c}}}{{\text{a}}}} \right)$ is the stretching ($\lambda$ > 0) or shrinking ($\lambda$ < 0) parameter and $S$ is the *wall* *transpiration* *(lateral* *mass* *flux)* *velocity* *parameter* with $S > 0$ for suction and $S < 0$ for injection. The featured dimensionless thermal, nanoscale and bioconvection parameters are:

$${\text{Pr}} = \frac{{{\upnu }_{\infty } }}{{{\upalpha }_{\infty } }}\quad \left( {{\text{Prandtl }}\,{\text{number}}} \right)$$

$${\text{Le}} = \frac{{{\upnu }_{\infty } }}{{{\text{D}}_{{{\text{B}}\infty }} }}\quad \left( {{\text{Lewis }}\,{\text{number}}} \right)$$

$${\text{Nt}} = \frac{{{\tau D}_{{\text{T}}} \left( {{\text{T}}_{{\text{f}}} - {\text{T}}_{\infty } } \right)}}{{{\upalpha }_{\infty } {\text{T}}_{{{ }\infty }} }} = \frac{{{\tau D}_{{\text{T}}} \Delta {\text{T}}_{{\text{f}}} }}{{{\upalpha }_{\infty } {\text{T}}_{{{ }\infty }} }}\quad \left( {{\text{Thermophoresis }}\,{\text{parameter}}} \right)$$

$${\text{Nb}} = \frac{{{\tau D}_{{\text{B}}} \left( {{\text{C}}_{{\text{w}}} - {\text{C}}_{\infty } } \right)}}{{{\upalpha }_{\infty } }} = \frac{{{\tau D}_{{\text{B}}} \Delta {\text{C}}_{{\text{W}}} }}{{{\upalpha }_{\infty } }}\quad \left( {{\text{Brownian motion }}\,{\text{parameter}}} \right)$$

$${\text{Sc}} = \frac{{{\upnu }_{\infty } }}{{{\text{D}}_{{{\text{n}}\infty }} }}\quad \left( {{\text{Biconvection }}\,{\text{Schmidt }}\,{\text{number}}} \right)$$

$${\text{Pe}} = \frac{{{\text{bW}}_{{\text{c}}} }}{{{\text{D}}_{{{\text{n}}\infty }} }}\quad \left( {{\text{Bioconvection}}\;{\text{P}}\mathop {\text{e}}\limits^{\prime } {\text{clet}}\;{\text{number}}} \right)$$

21 $${\upsigma } = \frac{{{\text{n}}_{\infty } }}{{{\text{n}}_{{\text{w}}} - {\text{n}}_{\infty } }} = \frac{{{\text{n}}_{\infty } }}{{\Delta {\text{n}}_{{\text{w}}} }}\quad \left( {{\text{Bioconvection }}\,{\text{constant}}} \right)$$

The system of Eqs. (16–19), subject to the conditions (20) is a *seven-parameter,* *ninth* *order,* *multi-degree* *coupled* *and* *non-linear* *system* which describes the transport phenomena in boundary layer flow from the permeable stretching/shrinking sheet immersed in dilute nanofluid containing gyrotactic microorganisms. The relevant engineering design parameters are the gradients at the wall (sheet) i.e. *reduced* *skin* *friction* *coefficient,* *local* *Nusselt* *numbers,* *local* *Sherwood* *number* *and* *gradient* *of* *local* *density* *of* *motile* *micro-organisms*. These are defined mathematically as follows:

22 $${\text{Cfr}} = {\text{C}}_{{\text{f}}} {\text{Re}}_{{\text{x}}}^{{{ }\frac{1}{2}}} = {\text{f}^{{\prime\prime}}}\left( 0 \right),$$

23 $${\text{Nur}} = {\text{Nu}}_{{\text{x}}} {\text{Re}}_{{\text{x}}}^{{ - { }\frac{1}{2}}} = - {{\theta^{\prime}}}\left( 0 \right),$$

24 $${\text{Shr}} = {\text{Sh}}_{{\text{X}}} {\text{Re}}_{{\text{x}}}^{{ - { }\frac{1}{2}}} = - \phi^{\prime}\left( 0 \right),$$

25 $${\text{Nnr}} = {\text{Nn}}_{{\text{x}}} {\text{Re}}_{{\text{x}}}^{{ - { }\frac{1}{2}}} = - {{\chi^{\prime}}}\left( 0 \right).$$

Here *Re*_*x*_ is the local Reynolds number (= *ρu*_*e*_*(x)x/*$\mu_{\infty }$).

## Finite difference numerical solution with code blocks

Numerical solutions to the nonlinear ordinary differential equations (16–19) under conditions (20) are obtained with a centre-space finite difference method (FDM). The computational procedure comprises three stages:

(i) Discretization with a *finite* *difference* *method* with *central* *differencing*
(ii) Tridiagonal matrix manipulation
(iii) Iterative algebraic solution procedure.

This numerical method is described in detail in Na^47^. Of course, some key advantages of the numerical technique used are that the Code Blocks finite difference method is algebraically less cumbersome than other techniques such as homotopy analysis method (HAM) and Hartree’s differential difference method. It achieves very fast solutions, rapid convergence, excellent numerical stability and is easily programmed. In this method, the third and second order differential equations are first reduced to a system of nine first order equations. These are then discretized with central spaced finite difference equations approximations. These nonlinear algebraic equations are then linearized by Newton’s method and the matrix–vector form is obtained. The momentum Eq. (16) can be considered as a second order linear differential equation by setting $y\left( \eta \right) = f^{\prime}\left( \eta \right),$ $y^{\prime}\left( \eta \right) = f^{\prime\prime}\left( \eta \right),$ $y^{\prime\prime}\left( \eta \right) = f^{\prime\prime\prime}\left( \eta \right)$ where $f\left( \eta \right)$ is considered as a known functions. In this case Eq. (16) can be written as:

26 $$\left( {1 + {\text{c}}_{2} \phi } \right){\text{y}^{{\prime\prime}}} + \left( {{\text{c}}_{2} \phi^{\prime} + \frac{{{\text{m}} + 1}}{2}{\text{f}}} \right){\text{y}^{{\prime}}} - {\text{mf}}^{\prime}y + {\text{m}} = 0.$$

The resultant form is:

27 $${\text{P}}(\eta )y^{\prime\prime}(\eta ) + {\text{Q}}(\eta )y^{\prime}(\eta ) + {\text{R}}(\eta ){\text{y}}(\eta ) = {\text{S}}(\eta ).$$

Here the following notation applies:

28 $$\begin{aligned} & P\left( \eta \right) = \left( {1 + c_{2} \phi } \right) ,\,\,Q\left( \eta \right) = (c_{2} \phi^{\prime} + \frac{m + 1}{2}f), \\ & R\left( \eta \right) = - mf^{\prime\prime},\,\,S\left( \eta \right) = - m. \\ \end{aligned}$$

Equations (17, 18 and 19) i.e. the energy, nano-particle concentration and micro-organism density conservation equations are second order differential equations. The energy equation (17) can be re-written as:

29 $$(1 + {\text{c}}_{4} \phi ){\uptheta }^{\prime\prime} + \left( {{\text{Pr}}\frac{{{\text{m}} + 1}}{2}{\text{f}} + {\text{c}}_{4} \phi^{\prime} + {\text{Nb}}\left[ {1 + 2{\text{c}}_{6} \phi } \right]\phi^{\prime} + {\text{Nt}{\theta^{\prime}}}} \right){{\theta^{\prime}}} = 0.$$

Here:

$${\text{P}}\left( {\upeta } \right) = \left( {1 + {\text{c}}_{4} \phi } \right){ },\,\,{\text{Q}}\left( {\upeta } \right) = \left( {{\text{Pr}}\frac{{{\text{m}} + 1}}{2}{\text{f}} + {\text{c}}_{4} \phi^{\prime} + {\text{Nb}}\left[ {1 + 2{\text{c}}_{6} \phi } \right]\phi^{\prime} + {\text{Nt}{\theta^{\prime}}}} \right),$$

30 $${\text{R}}\left( {\upeta } \right) = 0,\,\,{\text{S}}\left( {\upeta } \right) = 0.$$

Similarly, the nanoparticle volume fraction (concentration) Eq. (18) can be written as:

31 $$\left( {1 + {\text{c}}_{6} \phi } \right)\phi^{\prime\prime} + \left( {{\text{Le}}\frac{{{\text{m}} + 1}}{2}{\text{f}} + {\text{c}}_{6} \phi^{\prime}} \right)\phi^{\prime} + \frac{{{\text{Nt}}}}{{{\text{Nb}}}}{{\theta^{\prime\prime}}} = 0.$$

Here:

32 $$P\left( \eta \right) = \left( {1 + c_{6} \phi } \right) ,\,\,Q\left( \eta \right) = \left( {Le\frac{m + 1}{2}f + c_{6} \phi^{\prime}} \right),\,R\left( \eta \right) = 0,\,S\left( \eta \right) = - \frac{Nt}{{Nb}}\theta^{\prime\prime}.$$

Finally, the *motile* *micro-organism* *equation* (19) can be written as follows:

33 $$\left( {1 + {\text{c}}_{8} \phi } \right){{\chi^{\prime\prime}}} + \left( {{\text{Sc}}\frac{{{\text{m}} + 1}}{2}{\text{f}} + {\text{c}}_{8} \phi^{\prime} - {\text{Pe}}\phi^{\prime}} \right){{\chi^{\prime}}} - {\text{Pe}}\phi^{\prime\prime}{\upchi } - {\text{Pe}{\sigma }}\phi^{\prime\prime} = 0.$$

Here:

$$P\left( \eta \right) = \left( {1 + c_{8} \phi } \right) ,\,\,Q\left( \eta \right) = \left( {Sc\frac{m + 1}{2}f + c_{8} \phi^{\prime} - Pe\phi^{\prime}} \right),$$

34 $$R\left( \eta \right) = - Pe\phi^{\prime\prime},\,\,S\left( \eta \right) = Pe\sigma \phi^{\prime\prime}.$$

This linear system is solved using the FORTRAN language with the help of Code Blocks software^46^. This is a *free* *C/C*++ and Fortran online environment designed to enable fast computation of user-defined mathematical models. It is highly extensible and fully configurable and easy to program across multiple platforms. Code Blocks can be extended with plugins. Any kind of functionality can be added by installing/coding a plugin and compiling and debugging functionality are extremely robust. In the finite difference code (FDC), the convergence criterion is taken as $10^{ - 5}$ and the asymptotic boundary conditions in Eq. (20) are replaced by using a value for the similarity variable $\eta_{max}$ as follows:

35 $$\eta_{max} = 6, \,f^{\prime}\left( 6 \right) = 1, \,\theta \left( 6 \right) = 0, \phi \left( 6 \right) = 0, \,\chi \left( 6 \right) = 0.$$

The effects of the governing parameters on the flow field and heat transfer characteristics are analyzed for both stretching and shrinking cases. The value of Prandtl number $Pr$ is fixed at 6.2 (water base fluid). We consider here, both cases of $m = 1$ and $1 < m$ which correspond to the stagnation-point flow from a *linearly* *stretching/shrinking* sheet. The choice of $\eta_{max} = 6$ ensured that all numerical solutions approached the asymptotic values correctly. Table 1 shows the comparison values of $f^{\prime\prime}\left( 0 \right)$*,* $- \theta ^{\prime}\left( 0 \right)$ *and* $\phi ^{\prime}\left( 0 \right)$ with those reported by Ibrahim et al*.*^52^ and Zaimi et al.^50^. The comparison is done by neglecting the existence of gyrotactic micro-organisms (by omitting Eq. (19) and setting $S = 0$ *and* $\lambda = 1$ in the boundary conditions (20)).

**Table 1:** Comparison of the values of $f^{\prime\prime}\left( 0 \right), - \theta^{\prime}\left( 0 \right) and - \varphi^{\prime}\left( 0 \right)$ with $S = 0, \lambda = 1$ in the boundary conditions (20) and taking $Pr = 1, Le = 2, Nt = Nb = 0.5, c_{2} = c_{4} = c_{6} = c_{8} = 0{ }$ and $m = 1$ in the models of Ibrahim et al*.*^52^ and Zaimi et al*.*^51^.

	FDM	ADM	Ibrahim et al.^52^	Zaimi et al.^51^
${\text{f}}^{\prime\prime}\left( 0 \right)$	$0$	0	$0$	$0$
$- {{\theta^{\prime}}}\left( 0 \right)$	$0.476745$	0.47676	$0.4767$	$0.476737$
$- {{\varphi^{\prime}}}\left( 0 \right)$	$1.045230$	1.04511	$1.0452$	$1.045154$

Generally close correlation is achieved between FDM and previous special case solutions in Refs.^51^ and ^52^.

## Validation with Adomian decomposition method (ADM)

Full corroboration of the *general* *bioconvection* *nanofluid* model defined by Eqs. (16)–(19) under boundary conditions (20) can only be achieved with a different computational or semi-numerical technique. This serves to add a dual confidence in the computations. An excellent technique known as the Adomian decomposition method (ADM) is employed to solve the boundary value problem. ADM^48^ uses a polynomial expansion method to achieve high accuracy computations. ADM is a very adaptive method and has been deployed extensively in recent years for nonlinear biological and nanoscale fluid dynamics problems including smart lubrication^53^, swirling Von Karman flows^54^, stagnation spin coating flows^55^ and electromagnetic biofluid pumping^56^. It features infinite series solutions and utilizes recursive relations. Applying ADM, we introduce.$L_{1} = \frac{{d^{3} }}{{d\eta^{3} }}(\,)$.and $L_{2} = \frac{{d^{2} }}{{d\eta^{2} }}(\,)$ with *inverse* *operators* defined as follows:

36 $$L_{1}^{ - 1} (\,) = \int\limits_{0}^{\eta } {\int\limits_{0}^{\eta } {\int\limits_{0}^{\eta } {(\,)d\eta d\eta d\eta } } }$$

37 $$L_{2}^{ - 1} (\,) = \int\limits_{0}^{\eta } {\int\limits_{0}^{\eta } {(\,)d\eta d\eta .} }$$

The unknown functions $f,\, \theta\, \phi\,and\,\chi$ are expressed as infinite series in Adomian polynomials of the form:

38 $$\left. \begin{array}{*{20}c} {f\left( \eta \right) = \sum \limits_{m = 0}^{\infty } f_{m} ,\theta \left( \eta \right) = \sum \limits_{m = 0}^{\infty } \theta_{m} ,} \\ {{\phi} \left( \eta \right) = \sum \limits_{m = 0}^{\infty } {\phi}_{m} ,\;\psi \left( \eta \right) = \sum \limits_{m = 0}^{\infty } \psi_{m} } \end{array} \right\}.$$

Here recursive formula is used to find all the components. The exact solutions are formulated as:

39 $$\left. \begin{array}{*{20}c} {f\left( \eta \right) = Lim\; \sum\limits_{m = 0}^{\infty } f_{m} \;,} \\ {\theta \left( \eta \right) = Lim\; \sum \limits_{m = 0}^{\infty } \theta_{m} \;,} \\ {\;\left( \eta \right) = Lim\; \sum \limits_{m = 0}^{\infty } \phi_{m} ,} \\ {\psi \left( \eta \right) = Lim\; \sum \limits_{m = 0}^{\infty } \psi_{m} \;} \end{array} \right\}.$$

**Figure 2:**
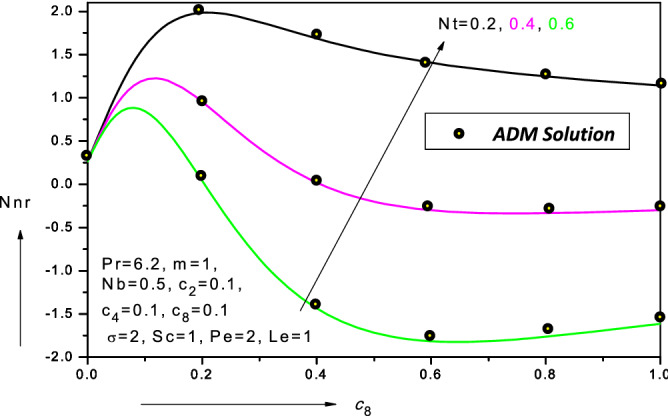
Variation of reduced density number of the motile micro-organisms (*Nnr*) with micro-organism diffusivity parameter ($$c_{8} )$$ in water-based nanofluid.

The algebraic expansions are lengthy and are therefore omitted here. The values of the similarity flow variables can be obtained readily. These may then in turn be utilized to compute the wall functions i.e. *skin* *friction,* *Nusselt* *number,* *Sherwood* *number* etc. Comparisons of the FDC solutions and the ADM code (which is executed on an SGI Octane desk workstation and takes approximately 100 s to converge) for *reduced* *micro-organism* *density* *number* *gradient* (*Nnr*) are presented in Fig. 2 (for the general model) for different values of thermophoresis parameter (*Nt*) and micro-organism diffusivity parameter ($$c_{8} )$$. The parameter ($$c_{8} )$$ features in Eq. (15) i.e. $$D_{n} \left( C \right) = D_{n,\infty } \left[ {1 + c_{7} \left( {C - C_{\infty } } \right)} \right] = D_{n,\infty } \left[ {1 + c_{8} \phi \left( \eta \right)} \right]{ }.$$ Clearly with increasing values of this parameter the mass diffusivity is increased. Although the effect is to initially elevate *Nnr* values, quickly they are depleted with subsequent elevation in micro-organism diffusivity parameter ($$c_{8} )$$. Generally higher micro-organism mass diffusivity will assist the propulsion of micro-organisms in the boundary layer away from the wall and will result therefore in a general decline in reduced micro-organism density number gradient (*Nnr*). With increasing thermophoresis parameter (*Nt*), a significant reduction is also computed in reduced micro-organism density number gradient (*Nnr*) is computed in Fig. 2. In all profiles, excellent correlation between the FDM and ADM solutions is achieved. Confidence in the FDM code is therefore justifiably high. Table 1 also shows the corroboration of FDM solutions for special cases with ADM.

## Results and discussion

**Figure 3:**
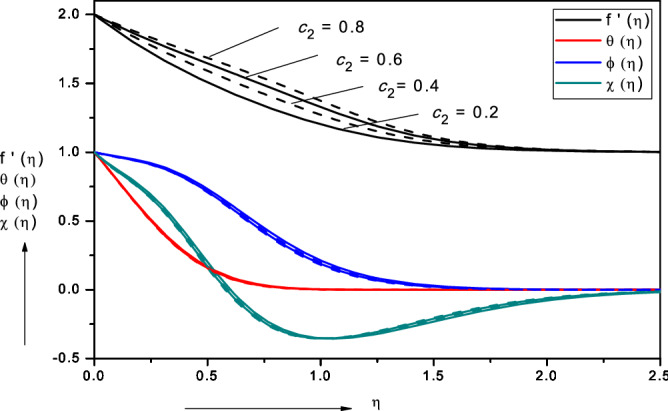
Effect of viscosity parameter ($c_{2}$) on nanofluid velocity, temperature, volume fraction and motile micro-organism density number.

**Figure 4:**
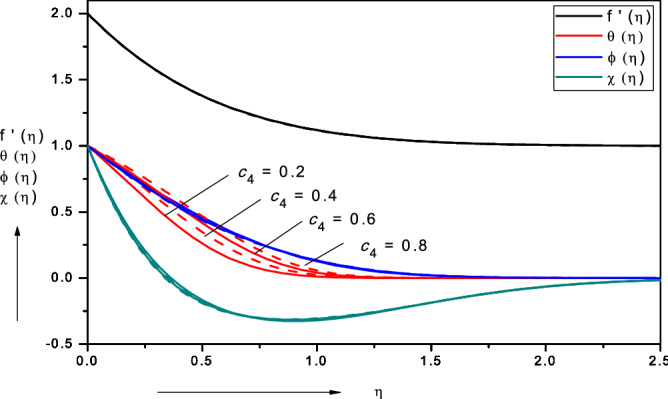
Effect of thermal conductivity parameter ($$c_{4} )$$ on nanofluid velocity, temperature, volume fraction and motile microorganism density number.

**Figure 5:**
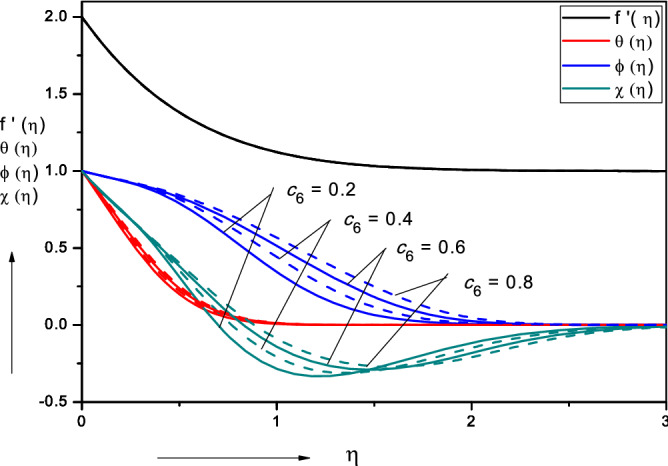
Effect of nano-particle mass diffusivity parameter ($$c_{6} )$$ on nanofluid velocity, temperature, volume fraction and motile micro-organism density number.

**Figure 6:**
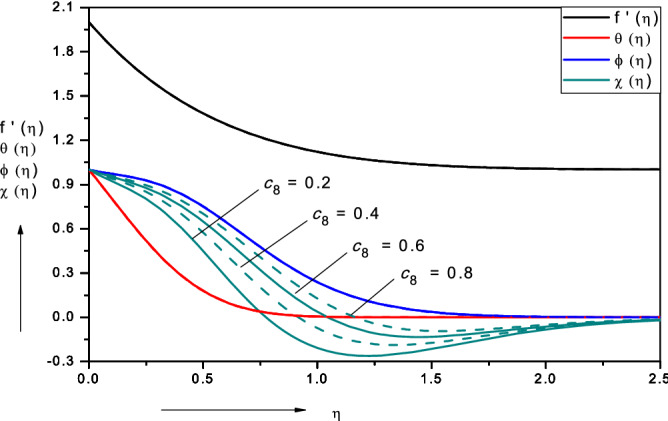
Effect of micro-organism species diffusivity parameter $\left( {c_{8} } \right)$ on nanofluid velocity, temperature, volume fraction and motile micro-organism density number.

**Figure 7:**
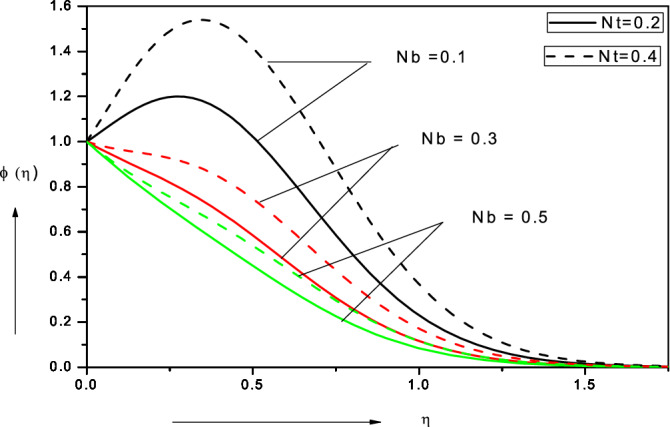
Effect of Brownian motion parameter ($Nb$) on nanoparticle volume fraction.

**Figure 8:**
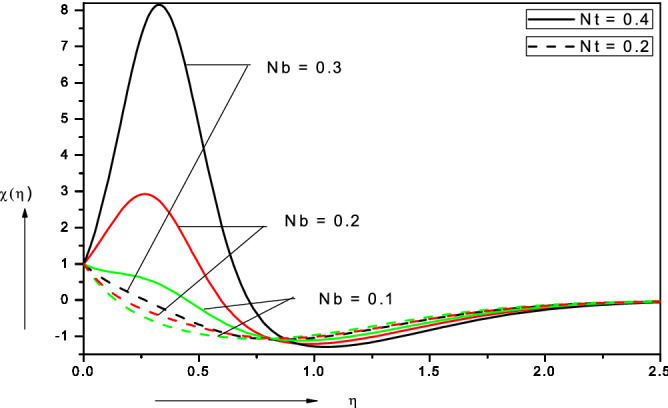
Effect of Brownian motion parameter ($Nb$) on motile microorganism density number.

**Figure 9:**
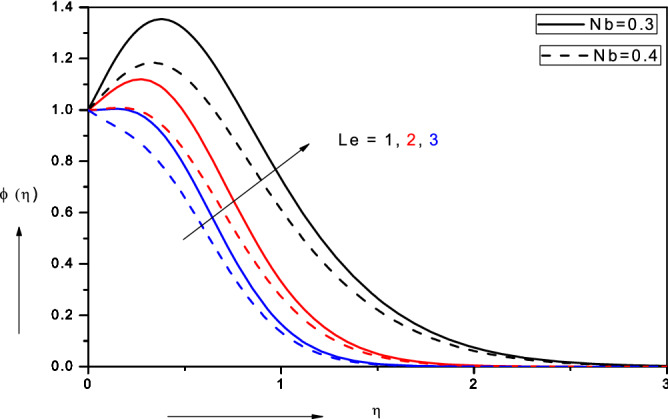
Effect of Lewis number ($$Le)$$ on nanofluid volume fraction.

**Figure 10:**
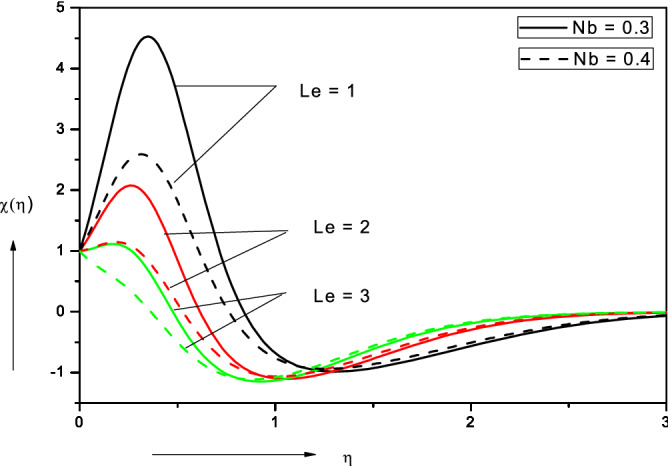
Effect of Lewis number ($$Le)$$ on nanofluid motile micro-organism density number.

**Figure 11:**
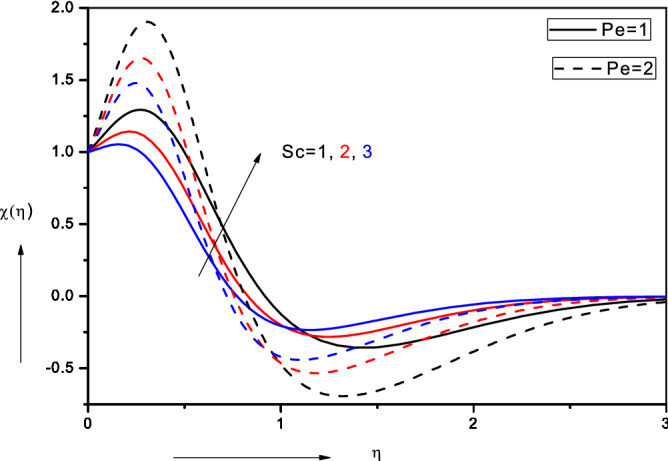
Effect of bioconvection Schmidt number ($Sc$) and bioconvection Péclet number $(Pe$) on motile micro-organism density number.

**Figure 12:**
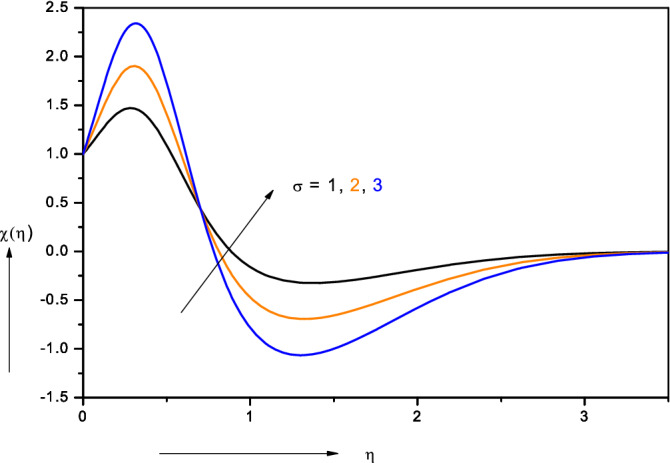
Effect of bioconvection constant ($\sigma )$ on motile microorganism density number.

**Figure 13:**
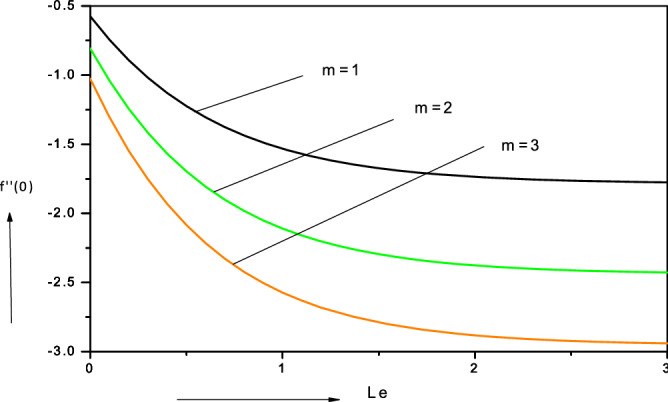
Variation of reduced skin friction coefficient with Lewis number ($Le$) in water-based nanofluid.

**Figure 14:**
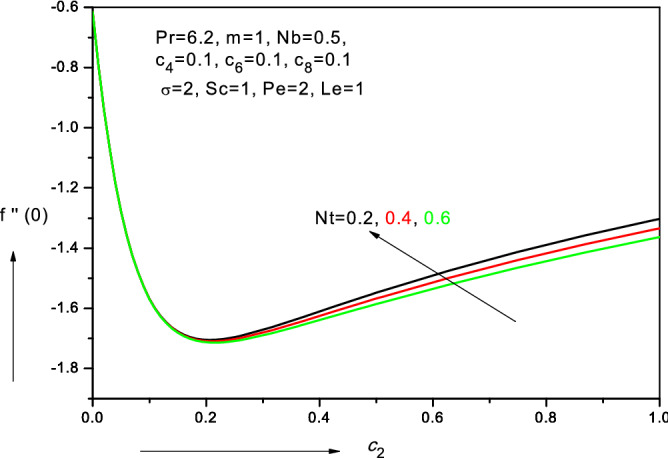
Variation of reduced skin friction coefficient with viscosity parameter ($$c_{2} )$$ in water-based nanofluid.

**Figure 15:**
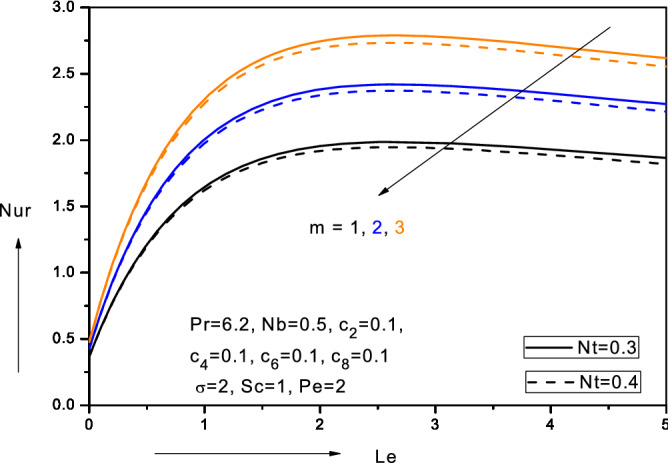
Variation of reduced Nusselt number (*Nur*) with Lewis number ($Le$), thermophoresis parameter (*Nt*) and velocity power-law index (*m*) in water-based nanofluid.

**Figure 16:**
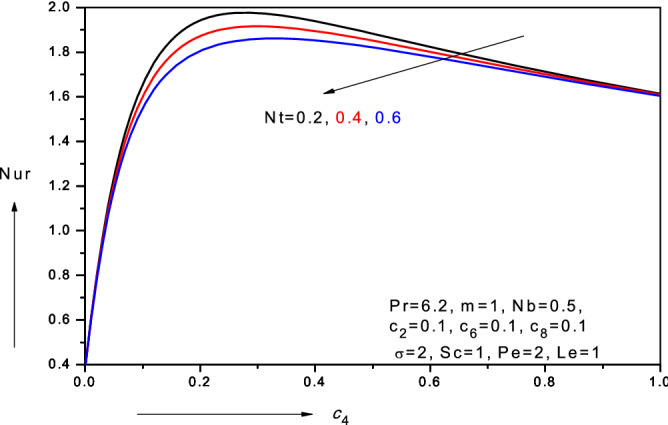
Variation of reduced Nusselt number (*Nur*) with thermal conductivity parameter ($$c_{4} )$$ and thermophoresis parameter (*Nt*) in water-based nanofluid.

**Figure 17:**
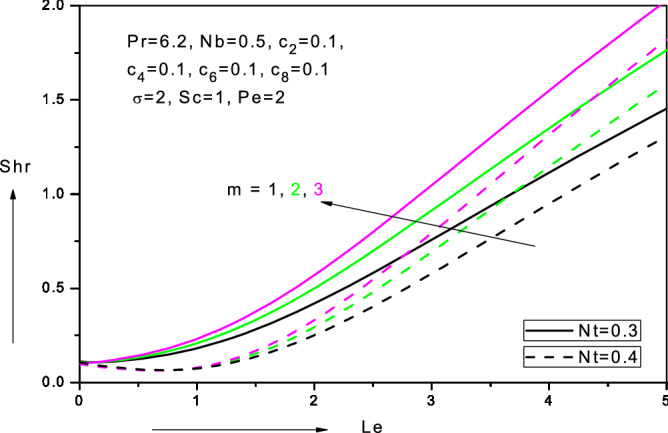
Variation of reduced Sherwood number (*Shr*) with Lewis number ($Le$), thermophoresis parameter (*Nt*) and velocity power-law index (*m*) in water-based nanofluid.

**Figure 18:**
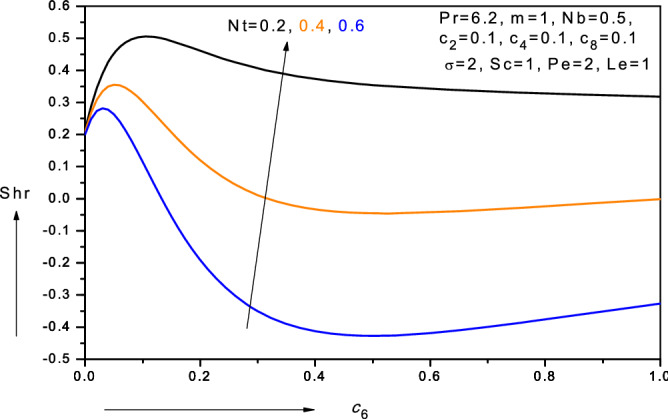
Variation of reduced Sherwood number (*Shr*) with nano-particle species diffusivity parameter ($$c_{6} )$$ and thermophoresis parameter (*Nt*) in water-based nanofluid.

**Figure 19:**
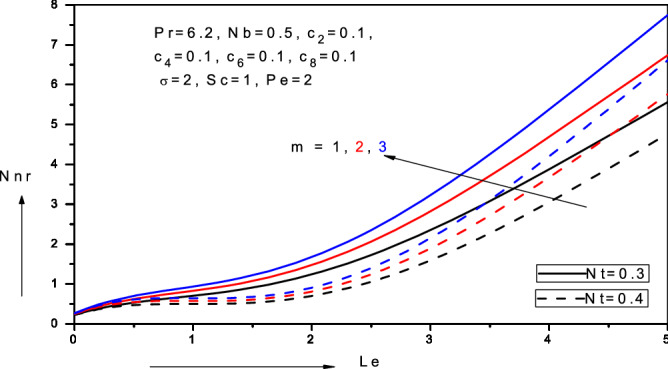
Variation of reduced density number of motile micro-organism (*Nnr*) with Lewis number ($Le$), thermophoresis parameter (*Nt*) and velocity power-law index (*m*) in water-based nanofluid.

Figures 3, 4, 5, 6, 7, 8, 9, 10, 11, 12 illustrate the FDM numerical results for dimensionless velocity $f^{\prime}\left( \eta \right),{ }$ temperature $\theta \left( \eta \right),{ }$ nanoparticles volume fraction, $\phi \left( \eta \right)$ and motile micro-organism density number, $\chi \left( \eta \right).$ The nonlinear boundary value problem is dictated by 11 controlling parameters i.e. $Pr, Le, Nt, Nb, Sc, Pe,$ $\sigma , c_{2} , c_{4} , c_{6}$, $c_{8}$. Since we have considered the base fluid as water $Pr = 6.2$, all remaining parameters are explored in the forthcoming figures. Figures 13, 14, 15, 16, 17, 18, 19 depict the evolution in local skin friction factor, $f{^{\prime\prime}}\left( 0 \right)$, reduced Nusselt number i.e. local heat transfer, $- \theta {^{\prime}}\left( 0 \right)$, reduced local Sherwood number $Shr$ i.e. local mass transfer, $- \phi {^{\prime}}\left( 0 \right)$ and reduced local density of the motile microorganisms $Nnr$ i.e. local micro-organism wall species gradient, $- {{\chi}} ^{\prime}\left( 0 \right)$ with various nanoscale, wall and bioconvection parameters.

### Effect of viscosity parameter ($$c_{2} )$$

Figure 3 illustrates the impact of *nanofluid* *viscosity* *parameter* $\left( {c_{2} } \right)$ on nanofluid velocity, temperature, volume fraction and motile micro-organism density number. Velocity is significantly enhanced with viscosity parameter increasing whereas temperature, volume fraction and motile microorganism density number are weakly increased. Since the parameter $\left( {c_{2} } \right)$ arises only in the transformed momentum Eq. (16) in the shear and diffusion terms, $\left( {1 + {\text{c}}_{2} \phi } \right){\text{f}}^{\prime\prime\prime} + {\text{c}}_{2} {\text{f}}^{\prime\prime}\phi^{\prime}$, the dominant influence is on the velocity field. However, there is direct coupling with the nano-particle species concentration field (*ϕ*) in these terms and furthermore both velocity and nano-particle concentration are coupled with multiple terms in the energy Eq. (18) and micro-organism species Eq. (20). These coupling terms include $(1 + c_{4} \phi )\theta^{{\prime\prime}} , + Pr\frac{m + 1}{2}f\theta^{\prime}, + c_{4} \theta^{\prime}\phi^{\prime}, + Nb\left[ {1 + 2c_{6} \phi } \right]\theta^{\prime}\phi^{\prime} + Nt\theta^{{\prime}{2}}$ in Eq. (17), $+ Le\frac{m + 1}{2}f\phi^{\prime}$ in Eq. (18) and $\left( {1 + c_{8} \phi } \right)\chi^{\prime\prime}, + Sc\frac{m + 1}{2}f\chi^{\prime}, + c_{8} \phi^{\prime}\chi^{\prime}and - Pe\left[ {\phi^{\prime}\chi^{\prime} + \phi^{\prime\prime}\left( {\sigma + \chi } \right)} \right]$ in Eq. (19). Evidently therefore there is an indirect influence of viscosity on temperature, nano-particle mass and micro-organism diffusion although the effect is relatively weak. Hydrodynamic (momentum) boundary layer thickness is therefore strongly reduced with viscosity parameter whereas thermal, nano-particle and micro-organism species boundary layer thicknesses are marginally increased. A similar observation has been reported by Begum et al.^45^. Asymptotically smooth profiles are computed in all cases indicating that a sufficiently large infinity boundary condition has been prescribed in the FDM code.

### Effect of thermal conductivity parameter $\left( {{\varvec{c}}_{4} } \right)$

Figure 4 illustrates the effect of thermal conductive parameter $\left( {c_{4} } \right)$ on nanofluid velocity, temperature, volume fraction and motile micro-organism density number. This parameter features exclusively in the energy conservation Eq. (17), in the terms, $(1 + c_{4} \phi )\theta^{^{\prime\prime}} , + c_{4} \theta^{\prime}\phi^{\prime}$. It is as with all variable properties a function of nano-particle volume fraction, ${\text{k}}_{\infty } \left[ {1 + {\text{c}}_{4} \phi \left( {\upeta } \right)} \right]$. With increasing thermal conductivity, the primary effect is to boost temperatures since molecular thermal conduction is assisted. A much weaker elevation in nano-particle concentration or micro-organism density number is recorded and again this is attributable to the weak indirect influence via coupling terms in the respective conservation equations. Velocity field is not tangibly influenced by changing thermal conductivity parameter. A significant increase in thermal boundary layer thickness is observed and thermal enhancement is confirmed, which concurs with numerous other studies including numerical results reported in Kang et al*.*^43^ and experimental findings described in Sahoo et al*.*^44^.

### Effect of nano-particle mass diffusivity parameter ($$c_{6} )$$

Figure 5 displays the impact of nano-particle species mass diffusivity parameter $\left( {c_{6} } \right)$ on nanofluid velocity, temperature, volume fraction and motile micro-organism density number. This parameter arises in the second order and first order terms, $\left( {1 + c_{6} \phi } \right)\phi^{\prime\prime}, + c_{6} \phi^{^{\prime}2}$ in the nano-particle concentration Eq. (18). The overwhelming effect is therefore to accentuate the diffusion of nano-particles in the boundary layer, as observed in the figure. Negligible modification in the velocity field is computed and a weak elevation in temperature field with increasing nano-particle species mass diffusivity parameter $\left( {c_{6} } \right)$. There is initially a substantial elevation in micro-organism density number for some distance into the boundary layer; however, with further progress towards the free stream this pattern is reversed and there is a depletion in micro-organism density number. The dominant influence of greater nano-particle species mass diffusivity parameter $\left( {c_{6} } \right)$ is to thicken the nano-particle concentration boundary layer thickness.

### Micro-organism diffusivity parameter $\left( {{\varvec{c}}_{8} } \right)$

Figure 6 illustrates the influence of micro-organism species diffusivity parameter $\left( {c_{8} } \right)$ on nanofluid velocity, temperature, volume fraction and motile micro-organism density number. This parameter occurs twice in the micro-organism concentration Eq. (19) in the terms, $\left( {1 + {\text{c}}_{8} \phi } \right){{\chi}}^{\prime\prime}$ and ${\text{c}}_{8} \phi^{\prime}{{\chi^{\prime}}}$. It is again linked to nano-particle volume fraction (*ϕ*) as per the definition in Eq. (15) i.e.${ }D_{n} \left( C \right) = D_{n,\infty } \left[ {1 + c_{7} \left( {C - C_{\infty } } \right)} \right] = D_{n,\infty } \left[ {1 + c_{8} \phi \left( \eta \right)} \right]$. Increasing micro-organism species diffusivity parameter $\left( {c_{8} } \right)$ generates a significant increase in the microorganism density number values (*χ*) at all values of transverse coordinate, *η*. However, at intermediate values of *η* as in other plots, negative values of micro-organism density number are computed. These correspond to a reversal in the direction of the swimming of the micro-organisms. Generally, however it is apparent that micro-organism boundary layer thickness is enhanced with greater values of the micro-organism species diffusivity parameter $\left( {c_{8} } \right)$. There is no substantial alteration in the velocity, temperature or micro-organism functions with increasing micro-organism species diffusivity parameter $\left( {c_{8} } \right)$.

### Brownian motion parameter (Nb) and thermophoresis parameter (Nt)

Figure 7 visualizes the evolution in nanoparticle volume fraction (*ϕ*) with various values of the Brownian motion parameter (*Nb*) and thermophoresis parameter (*Nt*).${ }Nb = \frac{{{\tau D}_{{\text{B}}} \left( {{\text{C}}_{{\text{w}}} - {\text{C}}_{\infty } } \right)}}{{{\upalpha }_{\infty } }} = \frac{{{\tau D}_{{\text{B}}} \Delta {\text{C}}_{{\text{W}}} }}{{{\upalpha }_{\infty } }}$ as defined in Eq. (21). It is a complex parameter and influenced by the concentration difference in nano-particles from the wall to the free stream among other effects. appears once in the energy conservation Eq. (17) in the term $+ Nb\left[ {1 + 2c_{6} \phi } \right]\theta^{\prime}\phi^{\prime}$ and also in the nano-particle concentration (volume fraction) Eq. (18) in the term,${ } + \frac{Nt}{{Nb}}\theta^{\prime\prime}$. In both cases it is coupled with the temperature function, *θ*. At low values of *Nb*, there is a distinct enhancement in nano-particle volume fraction with an associated jump (over-shoot) near the wall. However, with greater *Nb* values the nano-particle volume fraction is considerably reduced for all values of transverse coordinate. In the Buongiorno model the parameter *Nb* is inversely proportional to the size of nano-particles (which are assumed spherical and homogenously distributed in the base fluid). With greater *Nb* values smaller nano-particles are present and this intensifies the thermal conduction heat transfer from the particles to the surrounding fluid. This achieves the thermal enhancement which characterizes nanofluids as noted by Choi et al*.*^23^. Conversely however it stifles the molecular diffusion of nano-particles since smaller nano-particles are less successful in migrating through the base fluid and are more susceptible to ballistic collisions. Physically excessive concentrations of nano-particles (higher volume fractions) are counter-productive in nano-coating design and intermediate sized nano-particles have been shown to disperse more homogenously, as noted in Terentieva et al*.*^57^. There is a decrease in nano-particle concentration boundary layer thickness with larger values of the Brownian motion parameter. The overshoot near the wall is also eliminated at higher *Nb* values. The thermophoresis parameter (*Nt*) is the second major parameter featured in the Buongiorno nanoscale model^58^. As with the Brownian motion parameter, the thermophoresis parameter also occurs in both the energy conservation (17) and nano-particle volume fraction conservation (18) equation, specifically in the terms, $+ Nt\theta^{{\prime}{2}}$ and $+ \frac{Nt}{{Nb}}\theta^{\prime\prime}$. $Nt = \frac{{\tau D_{T} \left( {T_{f} - T_{\infty } } \right)}}{{\alpha_{\infty } T_{ \infty } }} = \frac{{\tau D_{T} \Delta T_{f} }}{{\alpha_{\infty } T_{ \infty } }}$ and is clearly a function of temperature difference across the boundary layer. Larger values of thermophoresis parameter, *Nt*, correspond to elevated migration of hot nano-particles in the direction of a *decreasing* *temperature* *gradient* which encourages nano-particle diffusion in the boundary layer. Thermophoretic forces exerted on the nano-particles are in the opposite direction to the actual temperature gradient. This effectively results in a boost in the diffusion of nano-particle species and a thicker nano-particle concentration boundary layer thickness. Therefore, thermophoresis induces the opposite response in the nano-particle concentration to that caused by Brownian motion. This pattern has also been identified in many other investigations including Kuznetsov and Nield^48^, Zaimi et al*.*^50^ and Ahmed et al.^59^. At high *Nb* and low *Nt* there is approximately a *linear* *decay* in nano-particle volume fraction from the wall to the freestream whereas at low *Nb* and high *Nt* a strongly *parabolic* profile is observed.

Figure 8 illustrates the collective influence of Brownian motion parameter (*Nb*) and thermophoresis parameter (*Nt*) on motile micro-organism density number (*χ*). Significant elevation in micro-organism density numbers is observed with rising Brownian motion parameter in the proximity of the wall. This behavior is however reversed further from the wall, although the subsequent decrease is much weaker than the initial enhancement. This may be attributable to the inverted boundary layer associated with wall stretching as noted by Zaimi et al.^50^ and also Amirsom et al*.*^51^. With greater thermophoresis parameter, *Nt*, there is also initially a significant elevation in motile micro-organism density number (*χ*) but again this trend is altered with subsequent penetration into the boundary layer i.e. as the free stream is approached. Clearly there is a complex relationship between nano-particle migration and micro-organism propulsion.

### Lewis number (Le)

Figure 9 shows the response in nanoparticle volume fraction (*ϕ*) to a modification in Lewis number (*Le*) and also Brownian motion parameter (*Nb*).${ }Le = \frac{{_{\infty } }}{{D_{B\infty } }}$ and this parameter arises in the single term, $+ Le\frac{m + 1}{2}f\phi^{\prime}$*,* which couples the thermal and nano-particle concentration boundary layers. Lewis number embodies the relative rate of heat diffusion to the nano-particle diffusion rate. It also expresses the relative thickness of the thermal and nano-particle concentration boundary layers. For *Le* = *1*, both boundary layers are of the same thickness and the diffusion rates are equal. For *Le* > 1 (of relevance in coating systems), the thermal diffusion rate exceeds the nano-particle diffusion rate and thermal boundary layer thickness is greater than nano-particle boundary layer thickness. There is therefore a significant reduction in nano-particle volume fraction with greater Lewis numbers, accompanied with a suppression in the near-wall overshoot which vanishes for *Le* = 3. This behavior is sustained throughout the boundary layer regime transverse to the surface of the stretching sheet (coating). Nano-particle concentration boundary layer thickness is therefore markedly depleted with greater Lewis number. Similarly increasing Brownian motion parameter, *Nb*, is also observed to suppress nano-particle concentration magnitudes and will lead to a thinner boundary layer thickness. Lewis number overall is a critical parameter determining the nano-particle distribution in the regime and has been shown to be highly impactful in determining nano-coating homogeneity and constitution during manufacturing processes^59^.

Figure 10 depicts the distribution in motile micro-organism density number (*χ*) with a variation in Lewis number (*Le*) and also Brownian motion parameter (*Nb*). Again, a significant suppression is observed in motile micro-organism density number (*χ*) with both increasing Lewis number and Brownian motion parameter and overshoots are eliminated. However, unlike the nano-particle concentration distribution (Fig. 9), this suppression is confined to the near-wall regime. Further from the wall there is a weak enhancement in motile micro-organism density number (*χ*) with both increasing Lewis number (*Le*) and also Brownian motion parameter (*Nb*). Asymptotic convergence of all the motile micro-organism density number profiles is computed in the free stream.

### Bioconvection Schmidt number ($Sc$), bioconvection Péclet number $(Pe$) and biconvection constant (σ)

Figure 11 illustrates the impact of *bioconvection* *Schmidt* *number* ($Sc$) *and* *bioconvection* *Péclet* *number* $(Pe$) on motile micro-organism density number (*χ*). Both increasing *Sc* and *Pe* values result in a boost generally in motile micro-organism density number (*χ*), although the latter only induces this nearer the wall and further from the wall there is a depletion. $Sc = \frac{{\nu_{\infty } }}{{D_{n\infty } }}$ and defines the relative momentum diffusion rate to the micro-organism diffusion rate. Since we have considered *Sc* > 1, there is clearly a strong increase in micro-organism boundary layer thickness with higher values of *bioconvection* *Schmidt* *number.* *Sc* arises in $+ Sc\frac{m + 1}{2}f\chi^{\prime}$ in the micro-organism conservation Eq. (19), and clearly exerts a strong influence on the distribution of micro-organisms in the boundary layer. Bioconvection Péclet number features in the micro-organism conservation Eq. (19), in the terms, $- Pe\left[ {\phi^{\prime}\chi^{\prime} + \phi^{\prime\prime}\left( {\sigma + \chi } \right)} \right].$ Bioconvection Péclet number relates the rate of advection of micro-organisms driven by the flow to the rate of diffusion of micro-organisms under gyrotaxis. Ordinary Péclet number is customarily associated with convective heat transfer processes and usually defines the heat transport via convection to that via thermal conduction. In bioconvection, this parameter when sufficiently high has been shown to dramatically alter patterns of the motile micro-organism flow. The source of bioconvection originates from the *internal* *energy* *of* *the* *micro-organisms*. With greater swimming speed (higher bioconvection Péclet number), the micro-organisms propel faster, and this *eventually* *decreases* *their* *concentrations*. At lower bioconvection Péclet numbers the reverse effect is induced i.e. motility of the micro-organisms is inhibited and they move slower leading to higher and significantly more homogenous concentrations in the bulk fluid. Clearly since $Pe = \frac{{bW_{c} }}{{D_{n\infty } }}$, for a given chemotaxis constant, *Pe* is directly proportional to *W*_*c*_ (constant maximum cell swimming speed) and inversely proportional to *D*_*n∞*_ (the diffusivity of micro-organisms). For *Pe* > *1*, swimming motions will dominate species diffusivity of micro-organisms and this will lead to a reduction in density of motile micro-organisms. The converse behaviour would arise for *Pe* < *1*. This parameter can therefore be manipulated via the selection of *different* *micro-organisms* (bio-species) to achieve a different distribution in combination with different nano-particles, in for example, the constitution of nano-bio fluid coatings, leading to a change in eventual performance of the coating. In fact, bioconvection Péclet number is also the ratio of the *characteristic* *velocity* *due* *to* *gyrotactic* *swimming* to a *characteristic* *velocity* *due* *to* *random* *diffusive* *swimming*. Since the microorganisms are heavier than water, their up-swimming creates unstable density stratification. Micro-organism boundary layer thickness is generally effectively decreased with bioconvection Péclet number ($Pe$).

Figure 12 shows the influence of bioconvection constant ($\sigma$) on motile micro-organism density number (*χ*). $\sigma = \frac{{n_{\infty } }}{{n_{w} - n_{\infty } }} = \frac{{n_{\infty } }}{{\Delta n_{w} }}$ and this parameter arises in the terms, $- {\text{Pe}}\left[ {\phi^{\prime\prime}\left( {{\upsigma } + {\upchi }} \right)} \right]$ in the micro-organism species conservation boundary layer Eq. (19). With increasing bioconvection constant ($\sigma$) the micro-organism magnitudes are initially strongly enhanced close to the wall whereas they are depressed further towards the free stream. The bioconvection constant links the free stream density of micro-organisms to the density difference across the boundary layer. As this parameter increases, there is a larger density gradient across the boundary layer region which encourages the propulsion of micro-organisms from the wall into the bulk flow. This manifests in a boost in motile micro-organism density numbers (*χ*) near the wall. However, with further distance from the wall this effect is reduced and negative values of motile micro-organism density numbers (*χ*) are computed indicating reversal in the swimming direction. When the upper surface of the suspensions is too dense due to the gathering of micro-organisms, it becomes unstable and micro-organisms descend to intensify bioconvection. Returning up-swimming micro-organisms maintain this bioconvection pattern, as noted in Refs.^37,42^ and ^54^.

### Skin friction distributions ($f^{\prime\prime}\left( 0 \right))$

Figure 13 illustrates the distribution of skin friction with Lewis number (*Le*) for various velocity power-law exponent values (*m*). For all values of *m* there is a clear decay in skin friction with increasing Lewis number. For the *linear* *power-law* case, system $$\left( {m = 1} \right)$$ it is seen that maximum skin friction is achieved $$f{^{\prime\prime}}\left( 0 \right)$$. With *m* > 1 there is a progressive depletion in skin friction indicating that significant flow deceleration is induced for nonlinear power-law velocity behavior at the wall. Figure 14 shows that initially with increasing viscosity parameter ($$c_{2} )$$ there is a sharp depletion in skin friction whereas with subsequent increase in viscosity parameter ($$c_{2} )$$ this trend is reversed and a significant enhancement is observed in skin friction although it is more gradual than the initial reduction rate. With increasing thermophoresis parameter (*Nt*), the skin friction remains invariant initially at low values of viscosity parameter ($$c_{2} )$$. However, with subsequent increase in viscosity parameter ($$c_{2} )$$ there is a notable depletion in skin friction with greater thermophoresis parameter.

### Reduced local Nusselt numbers, $Nur\left( { - \theta^{\prime}\left( 0 \right)} \right)$

Figure 15 depicts the variation in local heat transfer, $- \theta {^{\prime}}\left( 0 \right)$ i.e. reduced local Nusselt number, $Nur$ versus Lewis number $(Le$) for different values of velocity power-law exponent values (*m*) and thermophoresis parameter (*Nt*). With greater *m* values, there is a significant boost in reduced local Nusselt numbers at all values of Lewis number. Heat transfer rate at the wall is therefore minimized for the *linear* case, $\left( {m = 1} \right)$ and maximized for the *strongly* *nonlinear* case $\left( {m > 1} \right).$ With greater thermophoresis parameter, there is weak decrease in reduced local Nusselt number, $Nur$. Since thermophoresis promotes nano-particle diffusion in the boundary layer, this leads to a migration in nano-particles from the wall and an associated reduction in reduced local Nusselt numbers, $Nur$. Figure 16 illustrates the variation of the local Nusselt numbers, $Nur$ versus thermal conductive parameter $\left( {c_{4} } \right)$ for different values of thermophoresis parameter $(Nt$). As $c_{4}$ increases $- \theta {^{\prime}}\left( 0 \right)$ initially increases sharply but subsequently reduces gradually. Thermal conductivity variation therefore induces a substantive change in heat transfer rate at the wall. Although initially there is no variation in reduced Nusselt number with increasing thermophoresis parameter $Nt$, for low values of thermal conductive parameter $\left( {c_{4} } \right),$ with subsequent increase in *c*_*4*_ values, there is a notable decrease in $- \theta {^{\prime}}\left( 0 \right)$ i.e. reduced Nusselt number with greater $Nt$ values.

### Reduced local Sherwood numbers, $Shr$ ($- \phi ^{\prime}\left( 0 \right)$)

Figure 17 shows the variations of the local nano-particle mass transfer rate, $- \phi {^{\prime}}\left( 0 \right)$ i.e. reduced local Sherwood numbers $Shr$ versus Lewis number $(Le$) for velocity power-law exponent values (*m*) and thermophoresis parameter (*Nt*). With increasing *Le* and *m* values there is a strong elevation in reduced local Sherwood numbers $Shr$. Nano-particle wall mass transfer rate is therefore minimal for the linear case $\left( {m = 1} \right)$ and maximum for the strongly non-linear case $\left( {m > 1} \right).$ $- \theta {^{\prime}}\left( 0 \right)$ increases as $Le$ increases. Conversely with increasing thermophoresis parameter (*Nt*), there is a depletion in local nano-particle mass transfer rate, $- \phi {^{\prime}}\left( 0 \right)$ i.e. reduced local Sherwood numbers $Shr$. Figure 18 displays the variations of the local Sherwood numbers $Shr$ versus nano-particle mass diffusivity parameter $(c_{6} )$ for different values of thermophoresis parameter $(Nt$). As $c_{6}$ increases there is initially a steep ascent in $- \phi {^{\prime}}\left( 0 \right)$ values; however subsequently the profiles morph and a steady descent ensues for all further values of increasing nano-particle mass diffusivity parameter $(c_{6} )$. With increasing thermophoresis parameter, $Nt$, there is a marked suppression in local Sherwood numbers $Shr$. Clearly stronger thermophoresis encourages nano-particle diffusion in the boundary layer and results in a reduction in nano-particle mass transfer rate to the wall i.e. $- \phi {^{\prime}}\left( 0 \right)$ decreases as $Nt$ increases.

### Reduced density number of motile micro-organism (Nnr)

Figure 19 presents the variations of the local micro-organism reduced density number (*Nnr*) i.e. micro-organism wall mass transfer rate, $- {^{\prime}}\left( 0 \right)$ with Lewis number $(Le$) for various velocity power-law exponent values (*m*) and thermophoresis parameter (*Nt*). With increasing *Le* and *m*, there is a significant and consistent enhancement in local micro-organism reduced density numbers (*Nnr*)*.* However, with greater thermophoresis parameter there is a substantial suppression in local micro-organism reduced density numbers (*Nnr*) which is amplified with increasing Lewis numbers.

## Conclusions

Motivated by simulating new emerging bio-inspired nanoliquid film coating manufacturing processes, a mathematical model has been described for stagnation point flow toward a stretching or shrinking sheet of liquid nano-biofilm containing spherical nano-particles and bioconvecting gyrotactic micro-organisms. Mathematical relations have been included for variable transport properties of the liquid (viscosity, thermal conductivity, nano-particle species diffusivity) and micro-organisms (species diffusivity). Via appropriate similarity transformations, a dimensionless ordinary differential boundary value problem has been derived for the transport characteristics of the nano-biofilm dynamics. This emerging coupled ordinary differential equation system has been solved computationally with appropriate boundary conditions at the wall and in the free stream, with a central space finite difference method in the CodeBlocks Fortran platform. Graphical plots for the distribution of reduced skin friction coefficient, reduced Nusselt number, reduced Sherwood number and the reduced local density of the motile microorganisms as well as the velocity, temperature, nanoparticle volume fraction and the density of motile microorganisms have been presented for the influence of wall velocity power-law index (*m*), viscosity parameter $\left( {c_{2} } \right)$, thermal conductivity parameter (*c*_*4*_), nano-particle mass diffusivity (*c*_*6*_), micro-organism species diffusivity (*c*_*8*_), thermophoresis parameter $\left( {Nt} \right)$, Brownian motion parameter $\left( {Nb} \right)$, Lewis number $\left( {Le} \right)$, bioconvection Schmidt number $\left( {Sc} \right)$, bioconvection constant (*σ*) and bioconvection Péclet number $\left( {Pe} \right)$. Validation of the solutions via comparison related to previous simpler models has been included. Further verification of the general model has been achieved with the Adomian decomposition method (ADM). The major deductions which can be made from the present computations can be summarized as follows:

(i) Skin friction is elevated (i.e. the flow accelerated and the momentum boundary layer thickness decreased) with greater viscosity parameter (${\text{c}}_{2} )$ whereas it is suppressed with greater Lewis number and thermophoresis parameter.
(ii) Temperatures are elevated (as are thermal boundary layer thicknesses) with increasing thermal conductivity parameter (${\text{c}}_{4} )$ whereas Nusselt numbers are decreased.
(iii) Nano-particle volume fraction (concentration) is enhanced with increasing nano-particle mass diffusivity parameter ($c_{6}$) whereas it is markedly reduced with greater Lewis number (*Le*) and Brownian motion parameter (*Nb*).
(iv) With increasing stretching/shrinking velocity power-law exponent ($$m),$$ skin friction is decreased whereas Nusselt number and Sherwood number are both elevated.
(v) Motile microorganism density is boosted strongly with increasing micro-organism diffusivity parameter (${\text{c}}_{8}$) and Brownian motion parameter (*Nb*) but reduced considerably with greater bioconvection Schmidt number (*Sc*) and bioconvection Péclet number (*Pe*).
(vi) With increasing thermophoresis parameter (*Nt*), there is a significant reduction in local micro-organism reduced density numbers (*Nnr*) and this is magnified with increasing Lewis numbers.
(vii) The computational results achieved with the finite difference method (FDM) are numerically stable and accurate and this technique has been found to be very appropriate for nonlinear stagnation thin film nano-bio coating flow simulations of relevance to achieving good film growth in bio-inspired nanotechnological manufacturing^60,61^.

The current investigation has considered *non-magnetic* nano-particles. Electromagnetic nanofluids^62^ feature “smart” characteristics and are responsive to external electrical and magnetic fields. These are currently also being investigated and it is anticipated that alternative nanoscale models such as the *Tiwari-Das* model may also be explored.

## Notation

$a$: Constant in the ambient fluid velocity
$$b$$: Chemotaxis constant
ADM: Adomian decomposition method
$c_{p}$: Specific heat at constant pressure
$c$: Constant in the stretching/shrinking velocity
$$c_{2}$$: Viscosity parameter
*c*_*4*_: Thermal conductivity parameter
*c*_*6*_: Nano-particle mass diffusivity
*c*_*8*_: Micro-organism species diffusivity
$C$: Nano-particle concentration (volume fraction)
$Cfr$: Reduced skin friction
$C_{w}$: Uniform nanofluid volume fraction at the surface of the sheet
$C_{\infty }$: Uniform nanofluid volume fraction in the free stream
$D_{B} \left( C \right)$: Variable mass diffusivity of nanoparticles (variable Brownian diffusion coefficient)
$D_{n} \left( C \right)$: Variable diffusivity of gyrotactic micro-organisms
$D_{T}$: Thermophoretic diffusion coefficient
$D_{B,\infty }$: Constant nano-particle mass diffusivity
$D_{n,\infty }$: Constant micro-organism diffusivity
*f*: Dimensionless stream function
*FDM*: Finite difference method
*g*: Gravitational acceleration
$k\left( C \right)$: Variable thermal conductivity
$k_{\infty }$: Constant thermal conductivity
*Le*: Lewis number
$L_{1}$: ADM polynomials third order differential operator $= \frac{{d^{3} }}{{d\eta^{3} }}$
$L_{2} =$: ADM polynomials second order differential operator = $\frac{{d^{2} }}{{d\eta^{2} }}$
*m*: Stretching/shrinking wall velocity power-law index
$n$: Density of motile gyrotactic micro-organisms
$Nb$: Brownian motion parameter
$N_{w}$: Uniform concentration (number density) of gyrotactic microorganisms at sheet surface
$N_{\infty }$: Uniform concentration (number density) of gyrotactic microorganisms at sheet surface
*Nt*: Thermophoresis parameter
*Nur*: Reduced Nusselt number
*Nnr*: Reduced motile microorganism gradient density function
*P*(*η*): FDM discretization function
*Pe*: Bioconvection Péclet number
*Pr*: Prandtl number
*Q*(*η*): FDM discretization function
*R*(*η*): FDM discretization function
*Re*_*x*_: Local Reynolds number
*S*: Wall transpiration parameter
*P*(*η*): FDM discretization function
*Sc*: Bioconvection Schmidt number
*Shr*: Reduced Sherwood number
$$T$$: Nanofluid temperature
$T_{w}$: Uniform temperature at the surface of the sheet
$T_{\infty }$: Uniform temperature in the free stream
$\left( {u, v} \right)$: Nanofluid velocity components along, transverse to the sheet surface in (*x,y*) directions
$u_{e} \left( x \right)$: Ambient fluid velocity (dimensional external fluid velocity at edge of boundary layer)
$u_{w} \left( x \right)$: Stretching/shrinking velocity of bio-nano coating sheet
$v_{w} \left( x \right)$: Wall transpiration (suction/blowing) velocity at the bio-nano coating sheet surface
$W_{c}$: Maximum cell swimming speed

## Greek

σ: Bioconvection constant
$\lambda$: Coating sheet stretching ($\lambda$ > 0) or shrinking ($\lambda$ < 0) parameter
$\rho_{\infty }$: Constant fluid density
$\mu \left( C \right)$: Variable dynamic viscosity
$\tau$: Ratio of effective heat capacity of the nanoparticle material to the heat capacity of the base fluid (water) i.e. $\left( {\rho c} \right)_{p} /\left( {\rho c} \right)_{f}$
$\mu_{\infty }$: Constant dynamic viscosity
*η*: Dimensionless transverse coordinate
$\theta$: Dimensionless temperature function
$\phi$: Dimensionless nano-particle volume fraction
$\chi$: Dimensionless motile gyrotactic micro-organism density number
$\psi$: Dimensional stream function
